# Short and Medium-term Outcomes of Omphalocele and Gastroschisis: A Survey from a Tertiary Center

**DOI:** 10.1055/s-0041-1736299

**Published:** 2022-01-29

**Authors:** Alexandra Tavares Marques, José Estevão-Costa, Henrique Soares, Ana Catarina Fragoso

**Affiliations:** 1 Department of Surgery and Physiology, Faculty of Medicine, University of Porto, Porto, Portugal; 2 Pediatric Surgery Department, Centro Hospitalar Universitário de São João, Porto, Portugal; 3 Department of Pediatrics, Division of Neonatology, Faculty of Medicine of Porto, Centro Hospitalar Universitário de São João, Porto, Portugal

**Keywords:** omphalocele, gastroschisis, anomalies, abdominal-wall defect, morbidity, onfalocele, gastrosquisis, anomalias, defeito da parede abdominal, morbidade

## Abstract

**Objective** To characterize and compare the outcomes of omphalocele and gastroschisis from birth to 2 years of follow-up in a recent cohort at a tertiary center.

**Methods** This is a retrospective clinical record review of all patients with gastroschisis and omphalocele admitted to the Neonatal Intensive Care Unit between January 2009 and December 2019.

**Results** There were 38 patients, 13 of whom had omphalocele, and 25 of whom had gastroschisis. Associated anomalies were present in 6 patients (46.2%) with omphalocele and in 10 (41.7%) patients with gastroschisis. Compared with patients with omphalocele, those with gastroschisis had younger mothers (24.7 versus 29.6 years; *p* = 0.033), were born earlier (36 versus 37 weeks, *p* = 0 *.006* ), had lower birth weight (2365 ± 430.4 versus 2944.2 ± 571.9 g; *p* = 0.001), and had a longer hospital stay (24 versus 9 days, *p* = 0.001). The neonatal survival rate was 92.3% for omphalocele and 91.7% for gastroschisis. Thirty-four patients were followed-up over a median of 24 months; 13 patients with gastroschisis (59.1%) and 8 patients with omphalocele (66.7%) had at least one adverse event, mainly umbilical hernia (27.3% vs 41.7%), intestinal obstruction (31.8% vs 8.3%), or additional surgical interventions (27.3% vs 33.3%).

**Conclusion** Despite the high proportion of prematurity, low birth weight, and protracted recovery, gastroschisis and omphalocele (without chromosomal abnormalities) may achieve very high survival rates; on the other hand, complications may develop in the first years of life. Thus, a very positive perspective in terms of survival should be transmitted to future parents, but they should also be informed that substantial morbidity may occur in the medium term.

## Resumo

**Objetivo** Caracterizar e comparar os desfechos do onfalocelo e gastrosquisis desde o nascimento até aos 2 anos de seguimento numa coorte recente de um centro terciário.

**Métodos** Este é um estudo retrospectivo em que foi feita uma revisão dos registos clínicos de todos os pacientes com gastrosquisis e onfalocelo que foram internados na unidade de cuidados intensivos neonatais, entre janeiro de 2009 e dezembro de 2019.

**Resultados** Identificamos 38 pacientes, 13 dos quais tinham onfalocelo e 25 dos quais tinham gastrosquisis. Anomalias associadas estavam presentes em 6 pacientes (46.2%) com onfalocelo e 10 (41.7%) com gastrosquisis. Comparativamente com os pacientes com onfalocelo, os pacientes com gastrosquisis tinham mães mais jovens (24.7 versus 29.6 anos; *p* = 0.033), nasceram mais precocemente (36 versus 37 semanas, *p* = 0.006), com menor peso ao nascimento (2,365 ± 430.4 versus 2,944.2 ± 571.9 g; *p* = 0.001), e o internamento teve uma duração mais longa (24 versus 9 dias, *p* = 0.001). A taxa de sobrevivência neonatal foi de 92.3% para o onfalocelo e 91.7% para a gastrosquisis. Trinta e quatro pacientes foram seguidos durante um tempo mediano de seguimento de 24 meses: 13 com gastrosquisis (59.1%) e 8 com onfalocelo (66.7%) apresentaram pelo menos um evento adverso, sobretudo hérnia umbilical (27.3% vs 41.7%), obstrução intestinal (31.8% vs 8.3%) ou intervenções cirúrgicas adicionais (27.3% vs 33.3%).

**Conclusão** Apesar da alta proporção de prematuridade, de baixo peso e de recuperação lenta, os gastrosquisis, assim como os onfalocelos (sem anomalias cromossómicas), podem ter uma taxa de sobrevivência muito alta; por outro lado, nos primeiros anos de vida, podem surgir complicações não desprezíveis. Assim, aos futuros pais pode ser transmitida uma perspectiva muito positiva em termos de sobrevivência, embora eles também devam ser informados de que pode ocorrer morbidade substancial no médio prazo.

## Introduction

Gastroschisis and omphalocele are the most common congenital abdominal wall defects, with a prevalence of ∼ 2 to 4 per 10,000 live births, respectively. 1 The etiology of these defects remains poorly understood. In contrast to omphalocele, the prevalence of gastroschisis has been increasing worldwide in recent decades. 1

Omphalocele is a midline abdominal wall defect in which varying amounts of bowel and liver, and occasionally other organs, protrude through the base of the umbilicus into a membranous sac. The intestines are generally morphologically and functionally normal. 2 Prognosis is determined mainly by the associated anomalies but also by the size of the defect and degree of liver exteriorization. 3 4 Omphalocele with co-occurring anomalies, which represents over 60% of cases of omphalocele, is associated with a high rate of pregnancy terminations and intrauterine fetal deaths and a significant rate of mortality in the neonatal period and at 1 year of age. 3 5 6 Infants with isolated omphalocele have the best survival rate. 5 6

Gastroschisis is as an abdominal wall defected, in which bowel and sometimes other abdominal organs herniate into the amniotic cavity without a membranous protective sac. The prognosis of infants with gastroschisis is primarily determined by the degree of intestinal injury. 2 7 In contrast to omphalocele, the bowel in patients with gastroschisis is usually damaged due to chronic exposure to amniotic fluid, which results in a varying period of bowel dysmotility after birth, requiring prolonged parenteral nutrition and hospitalization. 8 9 10 Associated anomalies are present in 8 to 34% of cases, most of them in the gastrointestinal tract. 8 11 Extraintestinal and chromosomal abnormalities or recognizable syndromes are rare. 8

Despite advances in prenatal diagnosis, surgical management and neonatal critical care over the last decades have resulted in improvement of survival rates of infants born with gastroschisis and omphalocele, the survivors are still at significant risk of short and long-term adverse outcomes. 3 12 These include growth delay, neurodevelopmental deﬁciencies, recurrent abdominal pain, feeding difficulties, pulmonary insufﬁciency, recurrent infections, umbilical revision for hernia or scar repair, bowel obstruction requiring surgical treatment, and other surgical problems. 3 13 Efforts have been made to identify prenatal risk factors associated with gastroschisis and omphalocele, 14 the optimal timing and route of delivery, 15 16 immediate *vs* staged repair, 17 18 and other factors that may be amenable to improve outcomes as well to reduce the treatment costs. However, many of these issues still lacks consensus. 7 8 10

The aim of the present study was to retrospectively review recent live births with gastroschisis and omphalocele, managed at our tertiary care center, regarding demographic features, associated anomalies, neonatal outcomes, and progress of the survivors until 2 years of age.

## Methods

This is a retrospective study of all patients with gastroschisis and omphalocele that were born alive and were admitted to the Neonatal Intensive Care Unit of Centro Hospitalar Universitário de São João (CHUSJ), a tertiary center in Portugal, between January 2009 and December 2019. Elective terminations, spontaneous miscarriages, and intrauterine fetal deaths were excluded from our study. The present study received approval from the ethics committee of CHUSJ.

All data were obtained from the medical records of patients. Maternal information included age, parity, and previous abortions. Obstetric data included diagnosis (if prenatal, gestational age) and complications (amniotic fluid volume alterations, intrauterine growth restriction, gestational diabetes, and other). For the purposes of this study, intrauterine growth restriction (IUGR) was deﬁned as birth weight below the 10 ^th^ percentile. Gestational age at birth, mode of delivery, gender, birth weight, resuscitation, meconium aspiration, APGAR scores, herniated organs, time and type of surgical repair, and other surgeries during the first hospitalization were also obtained.

The morbidity indicators evaluated were length of hospital stay, duration of parenteral nutrition, time to initiate enteral feeding, need of mechanical ventilation, ventilator duration, complications (intestinal occlusion, necrotizing enterocolitis, and sepsis among others), and discharge with parenteral nutrition. Data of co-occurring major anomalies were also collected. We defined a major congenital anomaly as an anomaly that create signiﬁcant medical problems for the patient or that require speciﬁc surgical or medical management. 19 20 Anomalies considered physiologic (e.g., patent foramen ovale), transient or minor, were excluded. The classification of omphaloceles in giant/large or small was based on the defect size and on the qualitative descriptions (“small,” “large” and “giant”) used by pediatric surgeons in surgical reports. Giant omphalocele was defined as a defect with a diameter greater than 5 cm and/or containing greater than 75% of the liver in the sac. 3 Complex gastroschisis was defined as gastroschisis associated with at least one of the following intestinal complications: atresia, volvulus, perforation, and necrosis.

The follow-up during the first 2 years of life focused on the following variables: presence of umbilical hernia, intestinal obstruction, surgical interventions, need for parenteral nutrition, need for supplemental oxygen, and anthropometric parameters (weight and length).

The outcomes of patients born with gastroschisis were compared with those of patients born with omphalocele. Statistical analysis was performed using the IBM SPSS Statistics for Windows, Version 27.0 (IBM Corp., Armonk, NY, USA). Continuous variables were expressed as mean (± standard deviation) or median (P25–P75), in normal or skewed distribution, respectively. Categorical variables were described by absolute (n) and relative frequencies (%). For comparisons, parametric (independent *t* -test) and non-parametric (Mann-Whitney U test), tests were used for continuous variables with normal or skewed distribution, respectively. The Fisher exact test was used for comparisons involving categorical variables. Statistical significance was set at 5%. This study is reported in line with the STROBE checklist for observational studies. 21

## Results

There was a total of 38 patients, 13 with omphalocele and 25 with gastroschisis. All but two (one with omphalocele and one with gastroschisis) were diagnosed prenatally. One newborn with gastroschisis was transferred to another hospital on the 6 ^th^ day of life and not subsequently followed-up at our institution.

Maternal and neonatal demographic data are summarized in Table 1 . Maternal age was significantly lower for gastroschisis. Although not reaching statistical significance, there was a higher proportion of primigravida mothers in the gastroschisis group. There was no significant difference between omphalocele and gastroschisis regarding mother's history of previous abortions either.

**Table 1:** Maternal and neonatal demographics

	Total ( *n* = 38)	Omphalocele ( *n* = 13)	Gastroschisis ( *n* = 25)	*P* -value
**Maternal age (years)**	26.4 ± 6.8	29.6 ± 5.8	24.7 ± 6.8	0.033
**Gestational age at diagnosis (weeks)**	20 [13–24]	21 [12–22]	19 [13–27]	0.803
**Primigravida**	25 (65.8%)	7 (53.8%)	18 (72%)	0.301
**Previous abortions**	10 (26.3%)	4 (30.8%)	6 (25%)	0.709
**Gestational age at delivery (weeks)**	36 [35–37]	37 [36–38]	36 [34–36]	0.006
**Mode of delivery**				
** vaginal**	2 (5.3%)	2 (15.4%)	0	0.111
** cesarean section**	36 (94.7%)	11 (84.6%)	25 (100%)	
**Gender**				
** Male**	23 (60.5%)	7 (53.8%)	16 (64%)	0.728
** Female**	15 (39.5%)	6 (46.2%)	9 (36%)	
**Birth weight (grams)**	2,563.4 ± 551.1	2,944.2 ± 571.9	2,365 ± 430.4	0.001

Values are given as mean ± SD, median [P25 − P75] or n (%).

There were 2 (5.3%) multiple pregnancies, both with twins, discordant for the defect (1 with gastroschisis and 1 with omphalocele). Abnormal amniotic fluid volume (oligohydramnios or polyhydramnios) and IUGR were present in 2 (15.4%) and 3 (23.1%) omphalocele pregnancies, respectively. For the 22 gastroschisis pregnancies with available fetal ultrasound information, 2 (9.1%) had abnormal amniotic fluid volume and 7 (31.8%) had IUGR.

Two patients with omphalocele were delivered vaginally, while the other patients were delivered by elective or emergency cesarean section. At birth, patients with gastroschisis presented a significantly lower gestational age than patients with omphalocele. This reflects the higher proportion of preterm deliveries associated with gastroschisis (80%) when compared with omphalocele (53.8%). Birth weight was significantly lower for patients with gastroschisis than for those with omphalocele. For both defects, there was a similar preponderance of males.

Visceral content exteriorized through the abdominal wall defect at birth and associated anomalies are shown in Table 2 . Associated anomalies were present in 6 patients (46.2%) with omphalocele and in 10 (41.7%) with gastroschisis. Two male patients with gastroschisis had balanced translocations (t [6;8] [q13; p23.1] and t [2/9]) that were not associated with any known phenotype, and, therefore, were not included in the analysis. The translocation t(6;8)(q13;p23.1) was likely associated with infertility since the mother had had 5 previous pregnancy losses. Anomalies in the gastrointestinal tract were found only in patients with gastroschisis, the majority being bowel atresias. Of the 6 patients (24%) with complex gastroschisis, 2 presented with bowel atresia only, 1 presented with bowel atresia and perforation, and the other 3 presented with bowel perforation either alone or with other complications that define complex gastroschisis.

**Table 2:** Viscera exteriorized and associated anomalies

	Total ( *n* = 38)	Omphalocele ( *n* = 13)	Gastroschisis ( *n* = 25)
**Viscera exteriorized**			
**Bowel (only)**	20 (52.6%)	6 (46.1%)	14 (56%)
**Liver (only)**	3 (7.9%)	3 (23.1%)	−
**Bowel + liver**	3 (7.9%)	3 (23.1%)	−
**Bowel + liver + stomach + spleen**	1 (2.6%)	1 (7.7%)	−
**Bowel + stomach**	5 (13.2%)	−	5 (20%)
**Bowel + bladder and/or gonads**	6 (15.8%)	−	6 (24%)
Associated anomalies, total *	16 (43.2%)	6 (46.2%)	10 (41.7%)
**Beckwith-Wiedemann syndrome**	2 (5.4%)	2 (15.4%)	−
**Gastrointestinal anomalies, total**	6 (16.2%)	−	6 (25%)
**Bowel atresia**	3 (8.1%)	−	3 (12.5%)
**Enteric duplication cyst**	1 (2.7%)	−	1 (4.2%)
**Meckel diverticulum**	2 (5.4%)	−	2 (8.3%)
**Cardiac anomalies, total**	3 (8.1%)	2 (15.4%)	1 (4.2%)
**VSD**	1 (2.7%)	1 (7.7%)	−
**ASD**	2 (5.4%)	1 (7.7%)	1 (4.2%)
**Int-IVC**	1 (2.7%)	1 (7.7%)	−
**Central nervous system anomalies, total**	4 (10.8%)	3 (23.1%)	1 (4.2%)
**ACC**	1 (2.7%)	1 (7.7%)	−
**Nasal glial heterotopy**	1 (2.7%)	1 (7.7%)	−
**Hydrocephaly**	1 (2.7%)	−	1 (4.2%)
**Microcephaly**	1 (2.7%)	1 (7.7%)	−
**Cryptorchidism**	3 (8.1%)	1 (7.7%)	2 (8.3%)
**Hydronephrosis**	1 (2.7%)	−	1 (4.2%)
**Syndactyly**	2 (5.4%)	−	2 (8.3%)

Abbreviations: ACC, agenesis of the corpus callosum; ASD, atrial septal defect; Int-IVC, interrupted inferior vena cava; VSD, ventral septal defect

Values are given as n (%)

* *n* = 24 for associated anomalies with gastroschisis due to missing information from 1 patient transferred to another hospital

All patients with omphalocele had a normal karyotype. Genetic syndromes were found exclusively in patients with omphalocele (2 patients diagnosed with Beckwith-Wiedemann syndrome). There was a third patient with omphalocele and multiple anomalies in several organs, including one hemivertebrae, several cardiac malformations (mesocardia, ASD, interrupted inferior vena cava with azygos continuation to superior vena cava), and several facial abnormalities associated with Duane type 1 syndrome. There were 4 omphaloceles considered giant (30.8%), 3 of which had no associated anomalies. No patient had rupture of the omphalocele sac. One patient with a small omphalocele presented with ileum stenosis at birth.

Most patients had surgical repair of the abdominal wall defect on the first day of life, regardless of the type of the defect. Only one patient with omphalocele had surgery on the second day of life. Primary closure was achieved in 86.8% of the patients ( Table 3 ). Two patients (1 with complex gastroschisis and 1 with giant omphalocele) required silo placement after failed primary repair due to abdominal compartment syndrome. Both have died. For the other 3 patients (1 patient with complex and 2 with simple gastroschisis, respectively) who underwent staged closure, the mean time with silo was 10.7 days. Two patients with large omphaloceles required the use of a polytetrafluoroethylene (PTFE) patch. Intestinal atresia was repaired either in a second operation after primary closure (1 patient with complex gastroschisis) or during secondary abdominal closure (1 patient with complex gastroschisis). Intestinal stenosis (1 patient with complex gastroschisis and 1 patient with omphalocele) was repaired during primary closure.

**Table 3:** Comparison of clinical outcomes between patients with omphalocele and gastroschisis

	Total ( *n* = 38)	Omphalocele ( *n* = 13)	Gastroschisis ( *n* = 25)	*P* -value
**Resuscitation at birth**	6 (15.8%)	3 (23.1%)	3 (12.0%)	0.392
**APGAR Score**				
** 1 ^st^ minute < 7 **	4 (10.5%)	2 (15.4%)	2 (8%)	0.595
** 5 ^th^ minute < 7 **	2 (5.3%)	1 (7.7%)	1 (4%)	1.000
**Type of surgery**				
** Primary closure**	33 (86.8%)	12 (92.3%)	21 (84%)	0.643
** Staged closure (silo)**	5 (13.2%)	1 (7.7%)	4 (16%)	
More than 1 surgery during hospital stay * , ¶	7 (18.9%)	2 (15.4%)	5 (20.8%)	1.000
Hospital stay (days) *	20 [11.5–31.5]	9 [5.5–17.5]	24 [15.8–36.8]	0.001
**Need for mechanical ventilation** **Ventilator duration (days)**	34 (89.5%) 3 [1.8–5]	11 (84.6%) 2 [1–3.5]	23 (92%) 4 [2–6]	0.595 0.052
Parenteral nutrition duration (days) *	16 [9–27.5]	8 [3–13.5]	22.5 [14–34.5]	< 0.001
Initiation of enteral feeding (days of life) *	9.5 [4.8–14.5]	4 [2–5.5]	12 [9.5–19.5]	< 0.001
**Complications during hospital stay Total** *				
** Sepsis**	8 (21.6%)	1 (7.7%)	7 (29.2%)	0.216
** Intestinal occlusion**	2 (5.4%)	0	2 (8.3%)	0.532
Neonatal death *	3 (8.1%)	1 (7.7%)	2 (8.3%)	1.000

Values are given as median [P25–P75] or n (%)

* *n* = 24 for gastroschisis due to missing information from 1 patient transferred to another hospital

¶ For omphalocele: silo placement after failed primary closure ( *n* = 1), inguinal hernia repair and orchidopexy ( *n* = 1). For gastroschisis: silo removal with or without intestinal atresia repair ( *n* = 3), surgery for adhesion-related bowel obstruction ( *n* = 1), surgical wound debridement ( *n* = 1), intestinal atresia repair ( *n* = 1)

Overall, patients with gastroschisis had a higher rate of complications during the hospital stay, as shown in Table 3 . Sepsis and intestinal occlusion were more frequent, but without statistical significance, in patients with gastroschisis. Necrotizing enterocolitis was not found in any of the patients. Gastroschisis was significantly associated with a longer duration of parenteral nutrition and longer time to initiate enteral feeding. Patients with gastroschisis were hospitalized twice as long as patients with omphalocele. One of the patients with complex gastroschisis (bowel perforation only), who had an associated enteric duplication cyst, underwent a massive bowel resection during the primary repair surgery and developed ultra *-* short gut syndrome. One patient with Beckwith-Wiedemann syndrome, who had been born preterm (33 weeks) and with bronchopulmonary dysplasia, was discharged on nasogastric feeds and supplemental oxygen.

The neonatal survival rate was 92.3% for omphalocele and 91.7% for gastroschisis. All the three deaths occurred in the neonatal period and involved preterm neonates. Two deaths were of patients with gastroschisis. One of them presented complex gastroschisis with bowel and bladder exteriorized; the patient underwent silo placement and died on day 3 of life. The second patient was born by cesarean section in another hospital and suffered perinatal asphyxia. The death occurred on day 16 of life and was related with severe hypoxic-ischemic encephalopathy. The third patient was a twin born with a giant omphalocele containing the bowel, stomach, liver, and spleen. After failed primary closure, a silo was applied, but the patient died on day 3 of life.

Thirty-four patients were followed-up, with a median follow-up time of 24 months (range 11–24), and the outcomes are summarized in Table 4 . Thirteen patients with gastroschisis (59.1%) and 8 patients with omphalocele (66.7%) had at least one adverse event during the follow-up. Ten patients (29.4%) needed further surgical interventions due to diverse comorbidities, and the details are given in Supplementary Table S1 of the supplementary material. Seven patients with gastroschisis (31.8%) suffered intestinal obstruction episodes, with adhesions being the cause in 3 of them. Of the 5 surviving patients with complex gastroschisis, 3 underwent surgery for bowel obstruction during the follow-up period. Only 1 patient with omphalocele (8.3%) had bowel obstruction, and it was caused by an intestinal volvulus. The patient with complex gastroschisis that had ultra *-* short gut syndrome remained hospitalized for 788 days due to several complications (intestinal obstruction episodes and catheter-related infections) and lack of family support. Although it was possible to make progress to oral feeding, it was not possible to wean parenteral nutrition; therefore, one serial transverse enteroplasty (STEP) procedure was performed at 10 months. Despite that, the patient continued to be dependent on parenteral nutrition with irregular enteral tolerance during the follow-up period of our study. The patient with Beckwith-Wiedemann syndrome and bronchopulmonary dysplasia received supplemental oxygen until 1 year of age.

**Table 4:** Adverse outcomes during the follow-up

	Total ( *n* = 34)	Omphalocele ( *n* = 12)	Gastroschisis ( *n* = 22)	*P* -value
**Patients with adverse outcomes**	21 (61.8%)	8 (66.7%)	13 (59.1%)	0.727
**Umbilical hernia**	11 (32.4%)	5 (41.7%)	6 (27.3%)	0.459
**Intestinal occlusion episodes**	8 (23.5%)	1 (8.3%)	7 (31.8%)	0.210
** Surgical interventions ¶**	10 (29.4%)	4 (33.3%)	6 (27.3%)	0.714
**Parenteral nutrition**	1 (2.9%)	0	1 (4.5%)	1.000
**Supplemental oxygen**	1 (2.9%)	1 (8.3%)	0	0.353

Values are given as n (%)

¶ The surgical interventions are listed in Supplementary Table S1

Anthropometric data at 2 years of follow-up was available for 7 patients with omphalocele and 15 patients with gastroschisis. Only 1 patient with omphalocele (patient 2 in Supplementary Table S1 ) that had been born with both weight and length below the 10 ^th^ percentile was still below that percentile at 2 years of age. Of the 15 patients with gastroschisis, 3 had both weight and length at birth below the 10 ^th^ percentile, 1 had birth length below the 10 ^th^ percentile, and 1 had birth weight below the 10 ^th^ percentile. Only 1 patient with gastroschisis had the length below the 10 ^th^ percentile at 2 years of age, indicating improvement in the growth between birth and follow-up for most children.

## Discussion

This study examines the detailed short and medium-term outcome of 38 live births with omphalocele and gastroschisis. This represents approximately an institutional prevalence of 10 cases per 10,000 live births; however, our center is a referral one for antenatal diagnosis and therapy; that figure is probably indicative of an estimated prevalence of 3 to 5 cases per 10,000 live births in the country, as reported by others. 5 22 The etiology of these anterior abdominal wall defects and the associated risk factors remain controversial. Young maternal age has been recognized as one of the strongest risk factors for gastroschisis. 1 14 Consistent with this, the mothers of fetuses with gastroschisis were markedly younger (average 24.7 years) than mothers with fetuses with omphalocele (29.6 years) in our cohort of patients. For omphalocele, both young and advanced maternal age have been identified as risk factors. 5 14

The majority of patients in our cohort of cases were delivered at preterm. Gastroschisis patients were born earlier and with lower birth weight than those with omphalocele, which is consistent with other studies. 11 23 All patients with gastroschisis and 84.6% of the patients with omphalocele were delivered by caesarean section. The observed rate for cesarean delivery was high, and this is mainly explained by the fact that a planned delivery in a tertiary care center, with access to appropriate neonatal and pediatric surgical services, is more readily achieved by caesarean section than by vaginal delivery. There is also a fear of some obstetricians of inducing birth dystocia or injury of the exposed abdominal viscera during vaginal delivery. 7 However, several studies failed to demonstrate that cesarean section is superior to vaginal delivery for neonates with omphalocele and gastroschisis. 7 22 24

The choice of the method to reduce the exteriorized organs and close the fascial defect depends on several factors, namely the size of the defect, patient stability, gestational maturity, and the presence of associated anomalies. Many surgeons recommend primary closure, when feasible, to be done within a few hours after birth. 8 This is also the preferred strategy at our center. Our primary abdominal closure rate of 86.8% is similar to other published rates. 4 23 25 Staged repair with silo placement may be preferable for the unstable neonate, large defects and when gastroschisis is associated with significant intestinal damage. 4 7 In our study, one patient with a giant omphalocele and one patient with a large and complex gastroschisis developed compartment syndrome after primary closure and were converted to a staged reduction. Both patients died. This highlights the difficulties in managing patients with large defects as there is lack of strong evidence for decision-making. According to Lee et al., 26 initial primary closure should never be attempted for giant omphaloceles, while other studies report that immediate closure with or without the use of a synthetic patch is possible. 4 27

The length of hospitalization was significantly longer for patients with gastroschisis than for patients with omphalocele. The factors that contributed to this difference included the later onset of bowel function in patients with gastroschisis, as indicated by their significantly longer time in parenteral nutrition and to initiate enteral feeding, compared with patients with omphalocele. Furthermore, although not reaching statistical significance, patients with gastroschisis were more likely to have complications during hospitalization, namely sepsis. Our findings are in line with other studies that have shown that patients with gastroschisis generally have a more complicated neonatal course than those with omphalocele. 11 This is related to the expected bowel dysmotility and deficient nutrient absorption associated with gastroschisis, while patients with omphalocele typically have a normal or less impaired bowel function after birth. 2 Therefore, a longer period of parenteral nutrition is generally required for infants with gastroschisis while they wait for the recovery of gut function. During this period, patients are at risk of having complications associated with the parenteral nutrition, namely catheter-related sepsis, and liver disease. 28 In our cohort of patients with gastroschisis, 24% had complex gastroschisis, which is in line with other studies. 29 These patients typically have worse outcomes, and our findings confirm this.

Patients with omphalocele are more frequently diagnosed with associated anomalies compared with those with gastroschisis. 7 11 However, in our cohort of patients, we found that the proportion of patients diagnosed with associated anomalies was similar in omphalocele (46.2%) and in gastroschisis (41.7%). Given that elective terminations and intrauterine fetal deaths were excluded from our study, the proportion of patients with omphalocele and associated anomalies is lower than otherwise would have been expected. This also explains the absence of omphalocele cases with chromosomal defects. Similarly, Heider et al. 30 found that by excluding elective terminations and spontaneous miscarriages, the rate of associated anomalies in a cohort of 36 cases of omphalocele was 31%. We found a pattern of anomalies associated with each defect similar to what is reported in the literature. 6 8 11 31 Patients with omphalocele were more likely to have genetic syndromes and central nervous system anomalies, while gastrointestinal anomalies were more commonly associated with gastroschisis.

Survival rates greater than 90% have been reported for gastroschisis. 12 32 Our data corroborates this ﬁnding. We also report a high survival rate (92.3%) for omphalocele, and this is explained by the absence of cases with abnormal karyotype and the high proportion of isolated omphaloceles (53.8%) in our cohort of cases. Previously, Marshall et al. 5 reported a survival rate of 92% for patients with isolated omphalocele, and this value decreased to 38.8% for patients with chromosomal anomalies. Furthermore, excluding one case with bronchopulmonary dysplasia, none of the other survivors with omphalocele had significant cardiopulmonary complications during hospital stay. Thus, our group of patients with omphalocele may represent a selected subset of cases with low postoperative morbidity and mortality.

Medium-term morbidity was evaluated through examination of the outcomes listed in Table 4 for a median follow-up of 24 months, and through evaluation of the anthropometric parameters at 2 years of age. Infants with omphalocele and with gastroschisis typically show an early growth delay that improves over time in most cases. 3 10 20 We also observed an improvement of growth parameters from birth to 2 years of age, such that only 2 patients (1 with omphalocele and 1 with gastroschisis) had length and/or weight below the 10 ^th^ percentile at 2 years of age.

None of the outcomes in Table 4 were significantly different between gastroschisis and omphalocele. A higher proportion of patients with gastroschisis (31.8% versus 8.3%) had bowel obstruction during the follow-up period, mainly due to intestinal adhesions. Adhesive small bowel obstruction is a frequent and serious complication in the first year of life, particularly in patients with gastroschisis. 12 33 Van Eijck et al. 33 reported a series of 55 patients with gastroschisis and 92 patients with omphalocele, in which 25% and 13%, respectively, developed adhesive small bowel obstruction. The higher propensity of gastroschisis for adhesive small bowel obstruction is postulated to be a consequence of the inflammatory reaction of the bowel serosa following prolonged exposure to the amniotic fluid, and the hypoperistalsis that characterizes the newborns with gastroschisis also favors adhesion formation. 33

One of the most severe complications of anterior abdominal wall defects is short gut syndrome, which is the most common cause of intestinal failure in children. 34 Bowel atresia and other gastrointestinal anomalies, midgut volvulus, or necrotizing enterocolitis can predispose patients with gastroschisis and omphalocele to short bowel syndrome. 7 10 This condition can be handled with pharmacologic interventions and intestinal lengthening procedures with the ultimate goal of avoiding intestinal transplantation. In our study, one patient with complex gastroschisis who developed ultra-short gut syndrome and underwent one STEP procedure at 10 months was still dependent on total parenteral nutrition at 2 years of age.

Umbilical hernia is a well-known complication following repair of congenital abdominal defects. 12 35 The presence of umbilical hernia, abdominal scars or the lack of an umbilicus is known to cause psychosocial stress in some children and their parents. 13 23 Henrich et al. 23 reported a series of 15 patients with omphalocele and 20 patients with gastroschisis in whom 20% and 15%, respectively, developed a ventral hernia. In our study, we found a high proportion of patients with omphalocele (41.7%) and gastroschisis (27.3%) who developed an umbilical hernia. An expectant approach may be considered as the hernia resolves spontaneously in many patients. 35 In our study, only one patient underwent hernia repair during the follow-up period. For gastroschisis, the most consistent risk factor for umbilical hernia is sutureless closure, 35 but this method is not used at our center. Hawkins et al. 17 compared primary closure with SILO closure and found an equivalent hernia rate between primary closure and longer duration of SILO reduction, with SILO placed for less than 5 days associated with the lowest incidence of ventral hernia. 17 Given that, in our study, a small number of patients (3 survivors) underwent secondary closure, we cannot take conclusions about the impact of the repair method on the development of umbilical hernia.

Our results support a hopeful information in terms of survival to be transmitted to future parents of a child born with gastroschisis or with omphalocele with a normal karyotype. However, during prenatal counseling, parents should be informed that neonates with gastroschisis and omphalocele are at risk for serious complications, including longer duration of parenteral feeding, presence of other anomalies requiring more urgent treatment (e.g., bowel atresia), need of reoperation and prolonged hospital stays. Furthermore, some survivors may experience a non-negligible morbidity in the first 2 years of life, as explained above.

There are some limitations to our study, which include those of any retrospective analysis, such as selection bias, potential confounders, incomplete or missing data, and unmeasured factors that could have changed the results. In addition, this study does not contemplate all the mortality commonly associated with omphalocele because we included only live births. The sample is relatively small, but it is from a single center with standardized procedures of care, allowing a comprehensive and detailed characterization of the natural history and outcomes of these abdominal wall defects.

## Conclusion

Our study presents a comprehensive overview of the management and outcome of live births with gastroschisis and omphalocele in a large tertiary center. The present study corroborates that, when compared with omphalocele, patients with gastroschisis had younger mothers, lower gestational age and weight at birth, and a more prolonged hospitalization course. Both gastroschisis and omphalocele (with no chromosomal abnormalities) were associated with survival rates greater than 90%; however, significant morbidity may occur in the medium term.
