# Supplementary material for: Short and Medium-term Outcomes of Omphalocele and Gastroschisis: A Survey from a Tertiary Center
Source: Rev Bras Ginecol Obstet. 2022 Jan 29;44(1):10–8. doi: 10.1055/s-0041-1736299 (PMC9948110; doi:10.1055/s-0041-1736299)
Supplement: Supplementary file 1 — Supplementary Material [file 10-1055-s-0041-1736299-s210068.pdf]

Table S1 Patients that underwent further surgical procedures during the first year of life (excluding the surgeries done in first hospitalization after birth)

	Type of defect	Surgical procedure	Associated anomalies
Patient 1	Large omphalocele	Removal of PTFE patch	—
Patient 2	Large omphalocele	Colectomy due to bowel obstruction caused by volvulus	Cardiac malformations: VSD, ASD, mesocardia and interrupted inferior vena cava with azygos continuation to superior vena cava); facial abnormalities associated with Duane type 1 syndrome; hemivertebrae
Patient 3	Large omphalocele	Inguinal herniorrhaphy	—
Patient 4	Small omphalocele	Surgical excision of nasal glial heterotopy	(Ileum stenosis); nasal glial heterotopy
Patient 5	Simple Gastroschisis	Laparotomy for bowel obstruction caused by adhesions	Meckel diverticulum; cryptorchidism
Patient 6	Complex Gastroschisis	Laparotomy for bowel obstruction caused by adhesions; orchidopexy	(Bowel perforation); Cryptorchidism
Patient 7	Simple Gastroschisis	Laparotomy for bowel obstruction caused by adhesions and intestinal malrotation; ventriculostomy; inguinal herniorrhaphy	—
Patient 8*	Complex Gastroschisis	Serial transverse enteroplasty (STEP)	(Bowel perforation); enteric duplication cyst; syndactyly
Patient 9	Simple Gastroschisis	Umbilical hernia repair	—
Patient 10	Complex Gastroschisis	Laparotomy for bowel obstruction caused by an internal hernia involving the transverse colon (17 months); Laparotomy and sigmoid colostomy (24 months)	(Bowel perforation and stenosis)

Intestinal injuries at birth not considered true anomalies are in parentheses.
This patient had meningitis and sepsis few weeks after the laparotomy and developed several complications related with the meningitis
*This patient remained hospitalized during 788 days after birth and this surgery was done at 10 months of age and therefore it was included in this table and Table 4 and not on “More than 1 surgery during hospitalization” in ►**Table 3**

 © 2022. Federação Brasileira de Ginecologia e Obstetrícia. All rights reserved.
